# Ten-year survival and recurrence of hepatocellular cancer

**DOI:** 10.20517/2394-5079.2019.013

**Published:** 2019-10-13

**Authors:** Tomoki Sempokuya, Linda L. Wong

**Affiliations:** 1 Department of Medicine, John A. Burns School of Medicine, University of Hawaii at Manoa, Honolulu, Hawaii, HI 96813 , USA.; 2 Division of Gastroenterology and Hepatology, University of Nebraska Medical Center, Omaha, Nebraska, NE 68198, USA.; 3 Department of Surgery, John A. Burns School of Medicine, University of Hawaii at Manoa, Honolulu, Hawaii, HI 96813 , USA.

**Keywords:** Hepatocellular cancer, 10-year survival, liver transplantation, hepatic resection

## Abstract

**Aim:**

Long-term survival after hepatocellular cancer (HCC) is difficult to achieve likely related to recurrence. This study aimed to identify factors that were predictive of 10-year survival after the diagnosis of HCC.

**Methods:**

In a prospectively collected database of 1374 HCC cases (1993–2019), we identified 70 patients who survived over 10 years regardless of treatment. We then identified 164 patients in the entire cohort who either had liver resection or transplant, and died before 10 years. Demographics, tumor characteristics, treatment, recurrence and treatment of recurrence were compared.

**Results:**

Of the 10-year survivors, 36 underwent transplant, 27 had liver resection and 7 patients had only locoregional therapy. Compared to the non-survivors, the 10-year survivors were younger and had fewer comorbidities or recurrence, smaller tumor size, lower AST, ALT, AFP, platelets, neutrophil-to-lymphocyte ratio. Multivariate analysis showed only age and diabetes to be negative predictors. Recurrence occurred in 24 survivors (34.3%) with mean time to recurrence with standard deviation 57.1 ± 42.6 months compared to 80 non-survivors (48.7%) with mean time to recurrence of 15.3 ± 14.8 months. For hepatic resection, 10-year survivors had longer time to recurrence compared to non-survivors (median: 31.3 months).

**Conclusion:**

Long-term survivors mostly occur after resection or transplant, but 10% of our cohort survived 10 years with only locoregional therapy. Underlying health status maybe an important predictor of 10-year survival for patients receiving liver resections. Recurrence of HCC occurs in both 10-year survivors and non-survivors, but later recurrence with aggressive treatment of the recurrence may allow for 10-year survival.

## INTRODUCTION

Hepatocellular cancer (HCC) is the fourth leading cause of cancer mortality in the world and the incidence and mortality has been increasing in the USA^[1,2]^. Survival for HCC has been prolonged by curative therapies which include liver transplantation, hepatic resection and ablation^[3]^. The overall 5-year survival for patients with HCC is quite dismal and estimated at 10%–12%, however this is improved in patients with localized HCC (30%) and those who undergo liver transplantation (70%–75%)^[4,5]^. Efforts have been made to promote early detection of HCC with surveillance programs as this can contribute to improved survival by allowing patients to qualify for these curative therapies^[6]^.

Despite these efforts and potentially curative therapies, recurrence occurs in about 54% of patients who undergo resection and 8%–17% of those who undergo liver transplantation^[7–10]^. Recurrences have been treated with repeat liver resections, salvage liver transplantation after resection and locoregional therapies, however these recurrences are likely responsible for compromised long-term survival. While much of the literature focuses on 5-year outcome, less is reported about longer term survival beyond 5 years. Late recurrence, which occurs after 5 years, has been described in patients after resection or transplant^[11–13]^. Others have suggested that underlying liver function as measured by albumin-bilirubin (ALBI) grade correlated with recurrence free survival^[14,15]^.

There are few studies that report or critically evaluate 10-year survival from HCC. This is often difficult as patients relocate, have other illnesses, are lost to follow-up or are no longer followed by the tertiary center that performed the curative therapy. This study reviews a 26-year experience of patients in Hawaii with HCC who have been followed by a group of physicians and the state’s only Liver Center and characterizes patients with at least 10-year follow-up from curative therapies. Specifically, we identified 10-year survivors and compared them to patients who received similar therapies who died before 10 years.

## METHODS

### Patients

Utilizing prospectively collected database of 1374 HCC patients from 1993 to 2019, there were 575 patients who had at least 10 years of follow up. We identified those patients who survived at least 10 years regardless of treatment. We then selected a comparison group of all patients who underwent liver transplantation or liver resection and did not survive 10 years. We excluded patients who had liver transplantation or resection who were still alive but did not have at least 10 years of follow up. This comparison group also excluded patients who had non-surgical therapies and did not survive 10 years as this was a large heterogeneous group of patients with more advanced HCC and/or severe cirrhosis who received locoregional therapy or supportive care. This database is based on Hawaii’s only tertiary liver center and liver transplant program and also includes patients from the American territories of the Pacific Basin. Approximately 60%–70% of the HCC patients in Hawaii were referred to this center and included in this database. The diagnosis of HCC was made histologically or based on contrast-enhanced computed tomography scan or magnetic resonance imaging with typical HCC features based on guidelines published by the american association for the study of liver disease^[3]^. This study was approved by the university of hawaii at manoa institutional review board.

### Data collected

We obtained demographic (age, sex and ethnicity), anthropometric information [height, weight and body mass index (BMI)], comorbidities, etiology of HCC, tumor size/characteristics, laboratory values, staging, therapeutic modalities, recurrence and survival information. Ethnicity was categorized as “Caucasian”, “Asian”, “Pacific Islanders”, and “Others”. Comorbidity data collected include diabetes mellitus, smoking status, hyperlipidemia, and hypertension.

Significant alcohol use was defined as at least 2 alcoholic beverages daily for 10 years. Positive smoking history included both past and present use of cigarettes. Laboratory values include creatinine, alanine aminotransferase (ALT), aspartate aminotransferase (AST), albumin, bilirubin, prothrombin time with international normalized ratio, platelet, neutrophil, lymphocytes, hepatitis B virus (HBV) and hepatitis C virus (HCV) serologies and pre-treatment alpha feto protein (AFP). We defined normal AFP as less than 20 ng/dL. Based on these values, we calculated model for End-Stage Liver Disease (MELD) score^[16]^, fibrosis-4 (FIB-4) index for liver fibrosis^[17]^, AST/platelet ratio index (APRI) and neutrophil-lymphocyte ratio (NLR). The American Joint Committee on Cancer (AJCC) staging system was incorporated with tumor size, number, and location of tumor. Tumor size was categorized by the largest diameter by ≥ 5 cm or < 5 cm. Status on underlying cirrhosis, rupture at presentation, and macrovascular invasion on imaging was also noted. Cirrhosis was determined with imaging when tissue was not available. Therapeutic modalities included liver transplantation, hepatic resection and locoregional therapies (radiofrequency ablation (RFA), cryosurgery, transarterial chemoembolization (TACE), percutaneous ethanol injection and yttrium90 transarterial radioembolization). Liver resection was performed on patients with Childs-Turcotte-Pugh (CTP) A and early B with CTP of 7 without ascites or encephalopathy. Patients who received liver transplantations had unresectable tumor who met Milan criteria^[18]^ or prior liver resection with recurrence of HCC which met Milan criteria. Single tumors size less than 6.5 cm that were down staged to meet Milan criteria was also evaluated for liver transplantation since 2007.

### Statistical analysis

Statistical analysis was conducted using statistical package for social services (SPSS) (version 23.0. IBM Corp., Armonk, NY, USA), R version 3.4.1 (The R foundation for Statistical Computing, Vienna, Austria) as well as EZR version 1.36 (Division of Hematology, Saitama Medical Center, Jichi Medical University, Japan^[19]^). Primary objective of this study is to elucidate the factors associated with 10-year survival. Secondary objective of this study is to elucidate management strategies for HCC recurrence that allows for long term survival. Comparison of binary variables were accomplished by chi-square test. Continuous variables were analyzed by T test to obtain mean, standard deviation (SD) and standard error of mean (SE). Likelihood ratio was calculated for binary variables. Nominal regression was used to create a regression model. Variables included in this model as followings: age was categorized as a binary variable with < 65-year-old and ≥ 65-year-old. Demographic data, etiology of HCC, BMI, comorbidity, AFP as binary variable (normal vs abnormal), tumor size as a binary variable, Milan criteria, rupture status, and therapeutic modalities. Multivariable regression model was also created to analyze these variables with *P* < 0.1 on univariate analysis. We also conducted differences between 10-year survivors and non-survivors on time to recurrence for transplant and hepatic resection. Kolmogorov-Smirnov test was used to test for normal distribution. Mann-Whitney U test was used to compare time to recurrence and recurrence free survival between 10-year survivors and non-survivors. *P* < 0.05 is considered as statistically significant.

## RESULTS

### Baseline characteristics

This study included 234 patients: 70 patients who survived 10 years after the diagnosis of HCC and 164 patients in the entire cohort who either had liver resection or transplant and died before 10 years. Baseline characteristics are shown in Table 1. In the entire cohort, mean age was 60.2 years (SD: 10.6) with 153 patients (65.4%) older than 65-year, 175 (74.8%) were male, and 126 (53.8%) had BMI over 25. Ethnic distribution was as follows: 160 (68.4%) were Asian, 42 (17.9%) were Caucasian, 24 (10.3%) were Pacific Islanders, and 8 (3.4%) were mixed or another ethnicity. The incidence of risk factors included: 36.8% had prior HCV, 27.4% had prior HBV, 34.6% has alcohol usage, and 12.8% had NASH/NAFLD. For comorbid conditions, 125 (53.4%) had a smoking history, 66 (28.2%) had diabetes, 49 (20.9%) had hyperlipidemia, and 121 (51.7%) had hypertension. For tumor characteristics, 107 (45.7%) had tumor size ≥ 5 cm, 106 (45.3%) had normal AFP, and 108 (46.2%) met Milan criteria for liver transplantation. For treatment, 64 (27.4%) received liver transplantation, 163 (69.7%) had resection, and 7 patients had only locoregional therapy. For each treatment modalities, age, BMI ≥ 25, HCV, hyperlipidemia, tumor rupture, and size ≥ 5 cm had statistically significant difference among curative therapies. Of note, six patients had salvage transplant. More than half of the patients who were transplanted received locoregional therapy prior to transplant.

### 10-year survivors *vs*. non-survivors

Tables 2 and 3 summarize the characteristics of 10-year survivors *vs*. non-survivors. There was no difference in ethnic distribution between the groups. As shown in Tables 2 and 3, 10-year survivors were younger and had a smaller tumor size and lower AFP, AST, ALT, platelets and NLR compared to non 10-year survivors. Univariate analysis showed that 10-year survivors were less likely to be age ≥ 65 years or to have diabetes, hypertension or tumors ≥ 5 cm [Table 4]. Multivariate analysis showed only age and diabetes to be predictive of survival. Of the 10-year survivors, 36 underwent transplant, 27 had liver resection and 7 patients had only locoregional therapy. We performed separate analysis for transplantation and hepatic resection to compare 10-year survivors and non-survivors. Details are shown in Tables 5 and 6. For liver transplantation, HCC found with surveillance, hypertension and recurrence were significantly different in the univariate analysis. However, in the multivariate analysis, only the presence of recurrence was predictive of not surviving 10 years. For liver resection, Age ≥ 65-year, Hepatitis B, BMI ≥ 25, diabetes, hypertension, and smoking status had significant difference between two groups on the univariate analysis. Only BMI ≥ 25 and smoking were predictive of not surviving 10 years in the multivariate analysis.

### Recurrence

Of the 10-year survivors, recurrence occurred in 24 patients (34.3%) with mean time to recurrence with SD, 57.1 ± 42.6 months days and 23 of these patients had treatment for their recurrence. In 164 non 10-year survivors, 136 had liver resection and 28 had liver transplant. Recurrence occurred in 80 patients of non 10-year survivors (48.7%) with mean time to recurrence of 15.3 ± 14.8 months and 61 (76.3%) had treatment of the recurrence. Recurrence rate was 23.4% after transplant, 50.9% after resection and 85.7% after just locoregional therapy. For the liver transplant patients, 73.3% of recurrences received the following treatments: resections-5, RFA-2, external radiation-2 and systemic therapy-2. In the patients who received liver resection, 80.7% of recurrences were treated with the following; RFA-19, systemic therapy-15, TACE-14, repeat resection-11, radiation-3, Yttrium-90 radioembolization-2, and cryotherapy-1. Thirty-five liver resection patients had more than one recurrence and received: chemotherapy-17, RFA-7, TACE-7, repeat resection-2 and Yttrium-90 radioembolization-1. Of the 7 patients who had only locoregional therapy, 5 patients had RFA and 2 patients had TACE as their initial treatment. One patient had RFA for a 1.0 cm lesion and died 14 years later from cardiac problems. The other 6 patients had recurrences 3–11 years after their initial LRT and had subsequent procedures. Predictors of recurrence included alcohol abuse, HCV, screenable diagnosis, symptoms at the diagnosis, size ≥ 5 cm, treatment modalities (transplantation: 23.4%, resection: 50.9%, LRT: 85.7%). Age ≥ 65-year, AJCC staging, hypertension, hyperlipidemia, normal AFP, ethnicity, tumor rupture, presence of single tumor, or vascular invasion were not significant predictors of 10-year survival. For transplantation, there was significant difference on tumor recurrence with 13.9 % had recurrence for 10-year survivors and 35.7% had recurrence on non-survivors (*P* = 0.05). However, hepatic resection did not have significant difference on recurrence (*P* = 0.92). There was no difference between 10-year survivors and non-survivors regarding treatment status of recurrence for both transplant and hepatic resection. For transplantation, time to recurrence did not have significant difference between 10-year survivors and non-survivors. However, hepatic resection had significant difference (*P* < 0.001) between 10-year survivors [median: 938, interquartile range (IQR): 730–2155] and non-survivors (median: 357, IQR: 155–514). There was significant difference (*P* > 0.001) between 10-year survivors (median: 4065, IQR: 2,678–5,762) and non-survivors (median: 453, IQR: 174–1315) for recurrence free survival.

## DISCUSSION

### Characteristics of 10-year survivors *vs*. non-survivors

Survival after HCC has generally been related to the therapies that patients receive and which therapy they receive is mainly dependent on tumor characteristics and underlying liver function. Because HCC is such a heterogeneous neoplasm, the underlying liver function is further influenced by etiology of liver disease and external factors such as alcohol, smoking, and metabolic factors. When all things were considered in this study, patients who survived 10 years after diagnosis were more likely to be younger. Ten-year survivors also had smaller tumor size and fewer of them exceeded 5 cm. They may also have better underlying liver function as evidenced by lower liver enzymes and higher platelet count however fibrosis markers (FIB-4 and APRI) did not seem to differ between survivors and non-survivors. Previous studies have suggested differences in long term survival based on etiology of chronic liver disease with a better prognosis in those with viral hepatitis B or C compared to those with NASH or ALD^[20]^. Others have shown that underlying liver function can prognosticate long term survival^[14]^. Wu *et al*.^[21]^, in an evaluation of 8450 HCC patients long-term, determined that 10-year survival was dependent on the number of lesions, the presence of cirrhosis, child pugh classification and the time elapsed before first recurrence or metastasis. In this study, however, proportion of previous HBV or HCV infection did not differ in 10-year survivors and non-survivors. Non-survivors were more likely to have metabolic factors of diabetes and hypertension. Obesity, smoking and alcohol use did not seem to differ between survivors and non-survivors. However, after multivariate analysis, 10-year survivors were younger and less likely to have diabetes. Hypertension, size ≥ 5 cm and meeting Milan criteria were no longer significant after multivariate analysis.

### Treatment modality and survival

This current study attempted to characterize all 10-year survivors as previous studies have described 10-year survival after a particular modality: resection, transplant or RFA. Our study showed long-term survivors mostly occur after resection or transplant, but 10% of our cohort survived long-term with only locoregional therapy. Baseline characteristics in these three groups differed because of requirements for each of the therapies. To avoid confounding factors and bias, we conducted separate analyses for liver transplantation and resection.

Selection of patients for liver transplantation varies depending on the transplant center but generally requires AJCC stage I or II and the absence of macrovascular invasion, tumor rupture, high AFP, morbid obesity and severe medical comorbidities. In our center, we specifically require patients to have BMI 35 or less and AFP < 1000 ng/mL. In this study, only recurrence was a predictor of 10-year survival after transplantation. Surveillance, hypertension were no longer significant after multivariate analysis.

Previous studies have suggested that 10-year survival after liver resection was primarily dependent on tumor characteristics. Zheng *et al*.^[22]^, in 212 patients who underwent liver resection for HCC, reported 23% 10-year survival and predictors of survival included tumors < 5 cm, solitary tumors and the absence of vascular invasion. However, more than 20% of 10-year survivors had microvascular invasion, poor tumor differentiation, AFP greater than 1000 ng/mL and tumor size greater than 10 cm. Long-term survival may also be influenced by surgical expertise, as Chapman *et al*.^[23]^ reported that centers with high volumes of resections for HCC had significantly improved 10-year survival after hepatic resection. In our study, however, the univariate analysis on hepatic resection suggested that Age ≥ 65, HBV, BMI ≥ 25, smoking, and diabetes were associated with 10-year survival. With the multivariate analysis, only lower BMI and smoking were predictive of non-survival and all of the other tumor characteristics and recurrence did not affect survival.

This study did not compare 10-year survivors versus nonsurvivors in locoregional therapy specifically because we had a large heterogeneous group of patients who underwent locoregional therapy. Previous studies have demonstrated 10-year survival after ablative therapies, but these have typically involved patients with small tumors. Chen *et al.*^[14]^ in 271 patients with BCLC stage 0 patients with tumors < 2.0 cm, reported a 56.4% 10-year survival. While Shiina *et al*.^[24]^ used a more liberal criteria of ablating up to 5 cm tumors and noted a 10-year survival of 27.3% in 1170 patients.

### Recurrence

Recurrence of HCC occurred in both 10-year survivors and non-survivors, but later recurrence with aggressive treatment may have allowed for 10-year survival. Zheng *et al*.^[22]^ in 212 patients who underwent liver resection showed that 77% of the short term survivors developed recurrence within 2 years while 42% of the 10 year survivors developed recurrence most of whom had intrahepatic recurrences that were treatable. In a study of 878 patients with HCC, Lee *et al*.^[25]^ reported a 19.8% recurrence after transplant compared to a 64.9% recurrence after resection and suggested that transplant may have a protective effect against late recurrence of early stage HCC. Risk factors for recurrence included multiple tumors, tumor size, histologic features (grade, extent, vascular invasion) and preoperative AFP. Our study also showed lower recurrence rate after liver transplantation compared to hepatic resection and the importance of tumor recurrence on 10-year survival, especially after transplantation. Later recurrence was also associated with 10-year survival after liver resection. Tumor size ≥ 5 cm was associated with recurrence probably because these patients were ineligible for liver transplantation. Unlike previous reports, our study also included detailed information on comorbidities and risk factors and we also found that alcohol consumption was a predictor of recurrence.

### Limitations

This study was limited in that it was a single center study in a unique and diverse patient population in the Pacific which may limit generalizability. Our population had a large proportion of Asian and Pacific Islanders compared to a typical US study. We also had a high proportion of noncirrhotic HBV-related HCC patients, who were more likely to be candidates for resection. Geographic isolation of the entire state and smaller remote islands may also have limited access to care in different ways than larger states or countries. Finally, we reported all-cause mortality, so it is unclear if the non-10-year survivors died from HCC related issue or another problem.

In conclusion, long-term survivors mostly occur after resection or transplant, but 10% of our cohort survived 10 years with only locoregional therapy. Recurrence of HCC occurred in both 10-year survivors and non-survivors, but later recurrence with aggressive treatment of the recurrence may have allowed for 10-year survival. Finally, long-term survival and recurrence after HCC may be influenced by other comorbidities such as diabetes, smoking and alcohol use which may affect both the tumor and the overall health of the individual but larger studies would be needed to further investigate this.

## Figures and Tables

**Table 1.** Baseline characteristics for all patients

	Locoregional therapy (*n = 7*)	Resection (*n* =163)	Liver transplant (*n* = 64)	*P* value	Total (*n* = 234)
Age ≥ 65 years (%)	2 (28.5)	75 (46.0)	4 (6.3)	< 0.001	153 (65.4)
Sex (Males) (%)	5 (71.4)	115 (70.6)	55 (85.9)	0.05	175 (74.8)
BMI ≥ 25(%)	5 (71.4)	73 (44.8)	48 (75.0)	0.002	126 (53.8)
Diabetes (%)	1 (14.3)	47 (28.9)	18 (28.1)	0.70	66 (28.2)
Hepatitis B (%)	1 (14.3)	68 (41.7)	21 (32.8)	0.48	64 (27.4)
Hepatitis C (%)	5 (71.4)	43 (26.4)	38 (59.4)	< 0.001	86 (36.8)
Hyperlipidemia (%)	1 (14.3)	41 (25.2)	7 (10.9)	0.04	49 (20.9)
Hypertension (%)	5 (71.4)	88 (54.0)	28 (43.8)	0.12	121 (51.7)
AJCC Stages				0.08	
Stage I	7 (100)	109 (66.9)	49 (76.6)		165 (70.5)
Stage II	0 (0)	17 (10.4)	13 (20.3)		30 (12.8)
Stage IIIa	0 (0)	1 (0.6)	0 (0)		1 (0.4)
Stage IIIb	0 (0)	11 (6.7)	0 (0)		11 (4.7)
Stage IIIc	0 (0)	5 (3.0)	2 (3.1)		7 (3.0)
Stage III NOS	0 (0)	1 (0.6)	0 (0)		1 (0.4)
Stage IV	0 (0)	18 (11.0)	0 (0)		18 (7.7)
Single tumor	7 (100)	131 (80.4)	51 (79.7)	0.42	189 (80.8)
Cirrhosis	7 (100)	68 (41.7)	63 (98.4)	< 0.001	138 (60.0)
Normal AFP (%)	3 (42.9)	74 (45.4)	29 (45.3)	0.99	106 (45.3)
Rupture (%)	0 (0)	18 (11.0)	0 (0)	0.01	18 (7.7)
Size ≥ 5 cm (%)	3 (42.9)	98 (60.1)	6 (9.4)	< 0.001	107 (45.7)
Vascular invasion (%)	0 (0)	6 (3.7)	2 (3.1)	0.86	8 (3.4)

BMI: body mass index; AJCC: American Joint Committee on Cancer; NOS: not otherwise specified; AFP: alpha feto protein

**Table 2.** Patient characteristics of 10-year survivors *vs*. non-survivors

	10-year survivors (*n* = 70)	10-year Non-survivors (*n* = 164)	*P* value
Age in years	55.5 ± 7.5	62.2 ± 11.0	< 0.001
Age ≥ 65 years (%)	12 (17.1)	69 (42.1)	< 0.001
Sex (Males)	57 (81.4)	118 (72.0)	0.17
BMI ≥ 30(%)	12 (17.1)	31 (18.9)	0.77
Diabetes (%)	9 (12.9)	57 (34.8)	0.001
Hypertension (%)	31 (44.3)	90 (54.9)	0.001
Hyperlipidemia (%)	11 (15.7)	38 (23.2)	0.24
Smoking (%)	35 (50.0)	90 (54.9)	0.56
Alcohol use (%)	27 (38.6)	54 (32.9)	0.50
Hepatitis B (%)	30 (42.9)	60 (36.6)	0.12
Hepatitis C (%)	28 (40.0)	58 (35.4)	0.62
AJCC stages			0.09
Stage I	56 (80.0)	109 (66.4)	
Stage II	11 (15.7)	19 (11.6)	
Stage IIIa	1 (1.4)	10 (6.3)	
Stage IIIb	0 (0)	7 (4.3)	
Stage IIIc	0 (0)	1 (0.6)	
Stage III NOS	0 (0)	1 (0.6)	
Stage IV	2 (2.9)	16 (9.8)	
Single tumor	58 (82.9)	131 (79.9)	0.73
Cirrhosis	51 (72.9)	87 (53.0)	0.01
HCC found with surveillance (%)	12 (17.1)	27 (16.5)	1.00
ASKIU/L)	64.3 ± 45.5	73.6 ± 58.9	0.03
ALT(IU/L)	60.5 ± 38.5	64.0 ± 52.4	0.03
Platelets ( × 10^3^/cc)	149.6 ± 77.3	190.5 ± 101.2	0.03
FIB-4	4.32 ± 3.16	4.11 ± 3.66	0.07
APRI	0.62 ± 0.63	0.57 ± 0.75	0.94
Creatinine (mg/dL)	0.88 ± 0.21	1.01 ± 0.59	0.03
Neutrophil/Lymphocyte ratio	2.33 ± 1.88	4.20 ± 3.50	0.002
MELD	9.10 ± 3.3	9.28 ± 3.2	0.62
AFP (mg/dL)	2479 ± 14,355	13787 ± 81,011	0.049

Numerical values expressed as ± standard deviation. BMI: body mass index; AJCC: The American Joint Committee on Cancer staging system; NOS: not otherwise specified; HCC: hepatocellular cancer; AST: aspartate aminotransferase; ALT: alanine aminotransferase; FIB-4: fibrosis-4 Index; APRI: AST/Platelet Ratio Index; MELD: Model for End-stage Liver Disease Score; AFP: alpha feto protein

**Table 3.** Tumor characteristics and treatment of 10-year survivors *vs*. non-survivors

	10-year survivors (*n* = 70)	10-year Non-survivors (*n* = 164)	*P* value
Mean tumor size (cm ± SD)	4.0 ± 2.4	6.7 ± 4.7	< 0.001
Tumor size > 5 cm (%)	23 (35.4)	84 (51.2)	0.01
Single tumor (%)	58 (82.9)	131 (79.9)	0.73
Rupture (%)	2 (2.9)	16 (9.8)	0.12
Vascular invasion (%)	0 (0)	8 (4.9)	0.14
Met Milan Criteria (%)	40 (57.1)	68 (41.5)	0.04
Treatment			< 0.001*
Transplantation (%)	36 (51.4)	28 (17.1)	
Resection (%)	27 (38.6)	136 (82.9)	
Locoregional therapy (%)	7 (10.0)	0 (0)	
Recurrence (%)	24 (34.3)	80 (48.8)	0.04

* There was also significant difference between transplantation and resection (*P* < 0.001). SD: standard deviation

**Table 4.** Predictors of 10-year survival (all patients)

	Univariate odds-ratio (95%CI)	Multivariate odds-ratio (95%CI)
Age ≥ 65	**0.29 (0.14–0.57)**	**0.33 (0.15–0.72)**
Sex (Males)	1.71 (0.86–3.41)	
Hepatitis B	1.30 (0.74–2.30)	
Hepatitis C	1.21 (0.68–2.15)	
Alcohol history	1.28 (0.72–2.29)	
NASH/NAFLD	0.43 (0.16–1.17)	1.07 (0.32–3.52)
HCC found with surveillance	1.05 (0.50–2.21)	
BMI ≥ 25	0.84 (0.48–1.50)	
BMI ≥ 30	0.84 (0.40–1.75)	
Smoking	0.81 (0.46–1.42)	
Diabetes mellitus	**0.28 (0.13–0.60)**	**0.28 (0.12–0.68)**
Hyperlipidemia	0.60 (0.29–1.27)	
Hypertension	**0.36 (0.20–0.66)**	0.66 (0.33–1.31)
Normal AFP	0.67 (0.38–1.18)	
Size ≥ 5 cm	**0.47 (0.26–0.84)**	0.52 (0.14–1.91)
Met Milan criteria	**1.86 (1.06–3.28)**	0.69 (0.20–2.41)
Rupture	0.27 (0.06–1.22)	0.36 (0.07–1.85)

Significant values are in bold. NASH: non-alcoholic steatohepatitis; NAFLD: Non-alcoholic fatty liver disease; HCC: hepatocellular cancer; BMI: body mass index; AFP: alpha feto protein

**Table 5.** Predictors of 10-year survival after transplant

	Univariate odds-ratio (95%CI)	Multivariate odds-ratio (95%CI)
Age ≥ 65	2.45 (0.24–25.0)	
Sex (Males)	3.0 (0.66–13.3)	
Hepatitis B	1.41 (0.49–4.10)	
Hepatitis C	0.53 (0.19–1.48)	
Alcohol history	0.92 (0.34–2.49)	
NASH/NAFLD	0.76 (0.14–4.08)	
HCC found with surveillance	**0.29 (0.09–0.98)**	0.29 (0.07–1.21)
BMI ≥ 25	0.71 (0.22–2.26)	
BMI ≥ 30	0.86 (0.27–2.74)	
Smoking	1.40 (0.52–3.78)	
Diabetes	0.37 (0.12–1.14)	0.51 (0.12–2.20)
Hyperlipidemia	1.04 (0.21–5.09)	
Hypertension	**0.32 (0.10–0.98)**	0.28 (0.06–1.34)
Normal AFP	0.55 (0.20–1.50)	
Size ≥ 5 cm	1.62 (0.28–9.58)	
Single tumor	1.13 (0.33–3.84)	
Recurrence	**0.29 (0.09–0.98)**	**0.19 (0.03–1.02)**

Significant values are in bold. NASH: non-alcoholic steatohepatitis; NAFLD: non-alcoholic fatty liver disease; HCC: hepatocellular cancer; BMI: body mass index; AFP: alpha feto protein

**Table 6.** Predictors of 10-year survival after hepatic resection

	Univariate odds-ratio (95%CI)	Multivariate odds-ratio (95%CI)
Age ≥ 65	**0.35 (0.14–0.88)**	0.27 (0.43–4.50)
Sex (Males)	0.99 (0.40–2.45)	
Hepatitis B	**2.35 (1.01–5.45)**	2.14 (0.75–6.10)
Hepatitis C	0.43 (0.139–1.32)	
Alcohol history	1.16 (0.48–2.79)	
NASH/NAFLD	0.20 (0.03–1.55)	
HCC found with surveillance	1.59 (0.53–4.76)	
BMI ≥ 25	**0.27 (0.10–0.72)**	**0.32 (0.10–1.02)**
BMI ≥ 30	0.36 (0.08–1.64)	
Smoking	**0.33 (0.13–0.80)**	**0.25 (0.09–0.74)**
Diabetes	**0.08 (0.01–0.57)**	0.15 (0.02–1.22)
Hyperlipidemia	0.82 (0.31–2.22)	
Hypertension	0.36 (0.15–0.85)	0.63 (0.22–1.82)
Normal AFP	0.65 (0.28–1.53)	
Size ≥ 5 cm	0.96 (0.41–2.22)	
Met Milan criteria	1.03 (0.43–2.49)	
Rupture	0.60 (0.13–2.78)	
Single tumor	1.09 (0.38–3.14)	
Vascular invasion	< 0.01 (O-inf)	
Recurrence	0.88 (0.38–2.00)	

Significant values are in bold. NASH: non-alcoholic steatohepatitis; NAFLD: non-alcoholic fatty liver disease; HCC: hepatocellular cancer; BMI: body mass index; AFP: alpha feto protein
